# Transcriptional and physiological adaptations in nucleus accumbens somatostatin interneurons that regulate behavioral responses to cocaine

**DOI:** 10.1038/s41467-018-05657-9

**Published:** 2018-08-08

**Authors:** Efrain A. Ribeiro, Marine Salery, Joseph R. Scarpa, Erin S. Calipari, Peter J. Hamilton, Stacy M. Ku, Hope Kronman, Immanuel Purushothaman, Barbara Juarez, Mitra Heshmati, Marie Doyle, Casey Lardner, Dominicka Burek, Ana Strat, Stephen Pirpinias, Ezekiell Mouzon, Ming-Hu Han, Rachael L. Neve, Rosemary C. Bagot, Andrew Kasarskis, Ja Wook Koo, Eric J. Nestler

**Affiliations:** 1 Department of Neuroscience, Friedman Brain Institute, New York, 10029 NY USA; 2 Department of Genetics and Genomic Science, Icahn Institute of Genomics and Multiscale Biology, New York, 10029 NY USA; 3 Department of Pharmacological Sciences, Icahn School of Medicine at Mount Sinai, New York, 10029 NY USA; 4 Department of Brain and Cognitive Sciences, Massachusetts Institute of Technology, Cambridge, MA 02139 USA; 5 Department of Psychology, McGill University, Québec, H3A 1B1 Montreal Canada; 6 Department of Neural Development and Disease, Korea Brain Research Institute, Daegu, 41068 Republic of Korea

## Abstract

The role of somatostatin interneurons in nucleus accumbens (NAc), a key brain reward region, remains poorly understood due to the fact that these cells account for < 1% of NAc neurons. Here, we use optogenetics, electrophysiology, and RNA-sequencing to characterize the transcriptome and functioning of NAc somatostatin interneurons after repeated exposure to cocaine. We find that the activity of somatostatin interneurons regulates behavioral responses to cocaine, with repeated cocaine reducing the excitability of these neurons. Repeated cocaine also induces transcriptome-wide changes in gene expression within NAc somatostatin interneurons. We identify the JUND transcription factor as a key regulator of cocaine action and confirmed, by use of viral-mediated gene transfer, that JUND activity in somatostatin interneurons influences behavioral responses to cocaine. Our results identify alterations in NAc induced by cocaine in a sparse population of somatostatin interneurons, and illustrate the value of studying brain diseases using cell type-specific whole transcriptome RNA-sequencing.

## Abstract (web summary)

While making up a small percentage of neurons in the nucleus accumbens, somatostatin interneurons may have important function in dopamine- and addiction-related behavior. Here, Ribeiro and colleagues show that somatostatin interneurons regulate behavioral responses to cocaine with physiological and transcriptomic changes.

## Introduction

A major challenge in neuroscience is to characterize the contributions of a single cell type to the transcriptional profile and functioning of a brain region of interest. This challenge is especially prominent for GABAergic interneurons, since they often account for small fractions of cells within a given brain region. Despite their scarcity, the role of somatostatin (SST)-expressing GABAergic interneurons in regulating cortical plasticity has been well established over the last decade and highlights the profound impact that a miniscule population of neurons can have on plasticity of neural circuits in vivo. For example, a reduced number of somatostatin interneurons in neocortical and hippocampal regions studied postmortem has been reported for several neuropsychiatric disorders and this reduction is proposed to underlie some common pathological changes in circuit function across numerous syndromes^1,2^. However, somatostatin interneurons in the nucleus accumbens (NAc), a critical brain reward region implicated in drug addiction, remain poorly understood due to the fact that these cells represent less than < 1% of all NAc neurons, which makes their isolation and study technically challenging^3^. As somatostatin interneurons in NAc, cerebral cortex, and hippocampus originate from the same *Nkx2.1*(+) embryonic lineage (medial ganglionic eminence)^4^, this cell type might be important in regulating NAc plasticity as seen for the other forebrain regions.

A predominating theory of somatostatin interneuron function is that they control the flow of information into and out of a brain region by rhythmically inhibiting—via GABA and somatostatin release—the excitability of the distal dendrites of principal neurons and thereby their bursting^5–7^. There is pharmacological evidence concerning somatostatin signaling in NAc and dorsal striatum, showing that locomotor responses to amphetamine require somatostatin receptor activation, and that somatostatin peptide infusion into these regions rapidly induces dopamine release via presynaptic facilitation^8–14^. However, it is unclear to what extent the gene expression and physiology of somatostatin interneurons in NAc are altered by cocaine and what role these changes play in altering behavioral responses to the drug.

Only recently has it been possible to assess cell type-specific transcriptional mechanisms in D1- and D2-type principal medium spiny neurons (MSNs) of NAc that regulate their physiological responses to psychoactive drugs such as cocaine^15^. Still, pooling tissue from multiple animals, small sample sizes, and a focus on whole striatum have confounded these studies. In addition, sequencing studies have focused primarily on protein-coding genes by performing poly-A selection prior to sequencing, which enriches libraries with mRNA transcripts. However, polyadenylated transcripts obtained from whole cells represent fully mature transcripts and fail to capture many regulatory transcripts required for gene regulation in mammalian cells. There is therefore a need to perform total (as opposed to poly-A selected) RNA-sequencing on brain tissue to identify genome-wide transcriptional changes induced by a psychoactive drug in a specific cell type of an individual mouse.

In this study, we first use optogenetics to determine the functional role of NAc somatostatin interneurons in regulating behavioral responses to cocaine. We find that stimulation of these cells potentiates the effects of cocaine, while blocking their activity attenuates cocaine’s effects on behavior. We next record from EYFP-tagged somatostatin interneurons in NAc slices from cocaine- or saline-treated mice and found that repeated cocaine administration decreases the intrinsic excitability of these neurons. To characterize transcriptional alterations that cocaine induces in these cells, we perform cell type-specific RNA-sequencing on FACS-isolated nuclei of somatostatin interneurons and identified 1100 DETs enriched for processes related to neural plasticity. Using this strategy, we identify the transcription factor JUND as a differentially expressed target gene that regulates cocaine action. Finally, we use cell type-specific viral-mediated gene transfer to show that JUND function in NAc somatostatin interneurons regulates a subset of behavioral responses to cocaine. Together, our results identify a cell type-specific transcriptional mechanism underlying cocaine-induced alterations in NAc.

## Results

### NAc somatostatin interneurons and cocaine-elicited behaviors

**Fig. 1:**
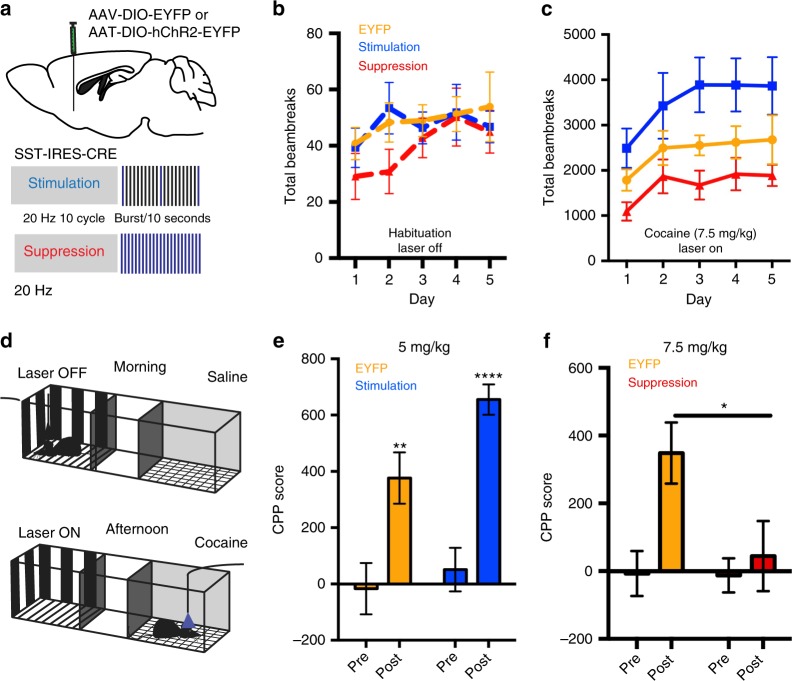
NAc somatostatin interneuron activity regulates cocaine-induced locomotor activity and reward. **a** AAV-DIO-EYFP or AAV-DIO-ChR2-EYFP were injected bilaterally into NAc of *Sst-Cre* mice 3 weeks prior to optogenetic stimulation or suppression, respectively, with the following parameters: Stimulation (473 nm, 20 Hz frequency, 1 burst of 10 pulses, 4 ms pulse width, every 10 s) or Suppression (473 nm, 20 Hz frequency, 1 burst of 20 pulses, 49 ms pulse width, every sec). **b** There is no difference between the locomotor activity of the three groups during habituation while the lasers remain off (repeated measures two-way ANOVA: no effect of activity F _(2,104)_ = 1.953. *p* = 0.147; *n* = 8,8,8) or day F_(4,104)_ = 0.3617. *p* = 0.9383; *n* = 8,8,8). **c** The activity of NAc somatostatin interneurons (EYFP control, Stimulation, or Suppression) controls the locomotor activity of mice after repeated cocaine injections. There is a significant effect of activity (repeated measures two-way ANOVA: significant effect of activity F_(2,21)_ = 5.647. *p* < 0.05; *n* = 8,8,8) and Day (Significant effect of Day F _(4,84)_ = 8.856. *p* < 0.001; *n* = 8,8,8). **d** Experimental design for unbiased CPP with optogenetics. Groups were counterbalanced to ensure that cocaine was paired evenly in both the gray and striped sides of the CPP chambers. **e** Stimulation of NAc somatostatin interneurons enhances cocaine CPP (Student's *t*-test: EYFP Pre-test vs EYFP Post-test, **p < 0.01; EYFP Pre-test mean = −16.50 ± 91.33 SEM vs EYFP Post-test mean = 376.4 ± 91.33 SEM (*n* = 8); CHR2-Stimulation Pre-test vs CHR2-Stimulation Post-Test, ****p < 0.0001 CHR2-Stimulation Pre-test mean = 51.00 ± 77.37 SEM vs CHR2-Stimulation Post-Test mean = 655.1 ± 53.93 SEM (*n* = 7); EYFP Post-test vs. CHR2-Stimulation Post-test, *p < 0.05 *n* = 8,7). **f** Cocaine CPP is blocked when NAc somatostatin interneurons are suppressed during training with cocaine. (Student’s *t*-test; AAV-DIO-EYFP Pre vs Post: **p < 0.01, EYFP Pre-test mean = −7.11 ± 66.2 SEM vs EYFP Post-test mean = 348.3 ± 90.41 SEM (*n* = 9)); AAV-DIO-CHR2 Pre vs Post: *P* > 0.05, *n* = 14). Data are represented as ± SEM

To investigate the functional role of NAc somatostatin interneurons in cocaine-elicited behaviors, we used optogenetics to bidirectionally control the activity of these cells in awake, freely moving mice (Fig. 1a). We injected an adeno-associated virus (AAV) vector expressing channelrhodopsin-2 (AAV-DIO-CHR2-EYFP)^16^, or EYFP alone as a control (AAV-DIO-EYFP), into NAc of adult *Sst-Cre* mice and allowed the vectors to express for 3 weeks. Immunohistochemistry for EYFP and SST protein confirmed the cell type-specificity of viral expression: all EYFP(+) cells also expressed SST (Supplementary Fig. 1). We validated the bidirectional optogenetic manipulation of NAc somatostatin interneurons in brain slices, achieving stimulation (473 nm, 20 Hz frequency, 1 burst of 10 pulses, 4 ms pulse width, every 10 s) versus suppression (473 nm, 20 Hz frequency, 1 burst of 20 pulses, 49 ms pulse width) of the neurons adapted from previous studies^17^ (Supplementary Fig. 2) (see Methods). Our finding that our suppression protocol completely silences ~50% of somatostatin interneurons and that the firing rate of the other half is reduced from 20 Hz to less than 4 Hz, is comparable to other groups’ findings using halorhodopsin or Designer Receptors Exclusively Activated by Designer Drugs (DREADDs) for cortical somatostatin interneurons^18,19^. Both groups achieved reduced action potential firing, but neither fully silenced somatostatin interneurons. Further validation of our stimulation and suppression protocols was obtained by C-FOS immunohistochemistry: we see >2-fold more C-FOS + MSNs after suppression of somatostatin interneurons than after their stimulation (Supplementary Fig. 2F–H). These results are consistent with the scheme that somatostatin interneurons control the excitability of principal neurons via distal dendritic inhibition in the striatum^20^.

We used this approach to first test the effect of stimulation or suppression of NAc somatostatin interneurons on cocaine-induced locomotor activation over 5 days. During each day of testing, the mice were allowed to habituate to the testing chambers for 5 min before laser stimulation commenced. Mice were injected with 7.5 mg/kg cocaine and locomotor activity was recorded for 30 min with simultaneous optogenetic stimulation or suppression. We found that NAc somatostatin interneurons bidirectionally control cocaine-induced locomotor activation (Fig. 1b, c), with stimulation of the cells promoting locomotor responses and suppression reducing such responses, findings that are in line with previous pharmacological studies^10,13^. We did not observe any differences in locomotor activity during habituation across the three groups, suggesting that the effects of NAc somatostatin interneuron activity are dependent on cocaine’s effects on the reward circuitry (see next section).

We proceeded to test the function of these cells on reward learning by use of conditioned place preference (CPP). In this experiment, we coupled optogenetic stimulation or suppression of NAc somatostatin interneurons with cocaine exposure, as previously performed in D1 and D2 MSNs^21^ (Fig. 1d). To study the effect of stimulating NAc somatostatin interneurons, we used a low dose of cocaine (5 mg/kg), as we hypothesized that this manipulation would increase the rewarding effects of the drug. Consistent with this hypothesis, AAV-DIO-CHR2-EYFP-injected mice developed a stronger CPP to cocaine than those injected with AAV-DIO-EYFP (Fig. 1e). Conversely, we used a higher dose of cocaine (7.5 mg/kg) to test the effect of suppressing NAc somatostatin interneuron activity and found that, while control mice formed a strong preference to this dose of cocaine, this effect was blocked upon suppression of the somatostatin cells (Fig. 1f). Together, these results establish a critical role for NAc somatostatin interneuron activity in controlling behavioral responses to cocaine.

### NAc somatostatin interneurons and baseline behavior

Given the finding that NAc somatostatin interneuron activity can modulate behavioral responses to cocaine, we determined if the same optogenetic manipulations alter locomotor or place conditioning behavior in the absence of cocaine. To do this, we stimulated or suppressed NAc somatostatin interneuron activity using optogenetics during 30 min of open field exploration. We found that neither optogenetic stimulation nor suppression alters locomotor activity compared to EYFP-expressing control mice. We repeated the experiment the next day and again found no difference between the groups (Supplementary Fig. 3A,C). Likewise, in separate groups of mice, neither optogenetic stimulation nor suppression of NAc somatostatin interneurons produced a CPP or aversion in the absence of cocaine (Supplementary Fig. 3B,D). We next tried to induce CPP using optogenetic stimulation or suppression of the neurons with a sub-threshold dose of cocaine (2.5 mg/kg) that did not produce CPP in control EYFP mice. Neither stimulation nor suppression of NAc somatostatin interneurons affected cocaine CPP at this lower drug dose (Supplementary Fig. 4). Together, these results show that somatostatin interneuron activity alone does not mimic the effect of cocaine on mouse behavior. Rather, these cells modulate behavior elicited by cocaine exposure.

### Cell type-specific RNA-sequencing of NAc somatostatin interneurons

**Fig. 2:**
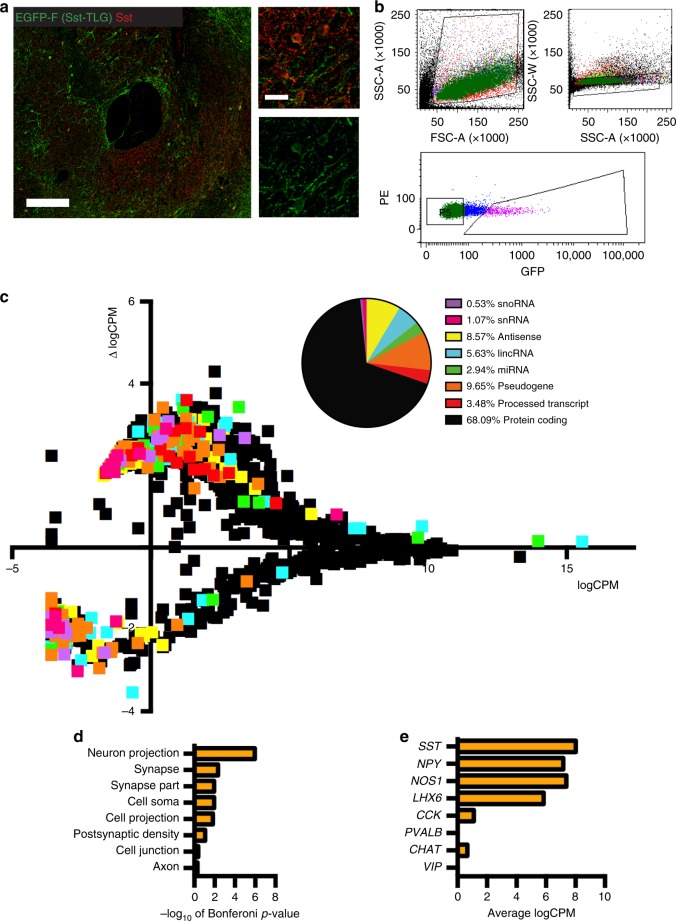
Cocaine induces 1100 DETs in NAc somatostatin interneurons. **a** Coronal section of anterior forebrain showing EGFP-F/SST overlap in NAc. Scale in **a** = 200 µm; Scale in inset = 25 µm. **b** Representative FACS gating used to isolate EGFP(+) nuclei from NAc of SST-TLG498 mice. We isolated ~5000 nuclei per mouse. **c** Differential expression analysis identifies 1100 cocaine-regulated transcripts in NAc somatostatin interneurons (*p* < 0.05). 10 mice were injected with saline and 10 with cocaine every day for 7 days. Mice were analyzed 1 h after the final injection and tissue was flash frozen and stored before nuclear FACS. On average, ~1–2 ng of RNA were collected per mouse as determined by analysis on RNA Pico Bioanaylzer chips (Agilent). 0.5–2 ng were then used to generate indexed libraries for sequencing using Clontech SMARTer Stranded Total RNA-seq library preparation kits. All libraries were analyzed for mean insert size and molar concentration prior to sequencing with V4 chemistry (Illumina) at Beckman Coulter Genomics (Now: GeneWiz). Samples were pooled in groups of 8 and all pools were sequenced across multiple lanes. We performed multiplexed Illumina HiSeq to obtain > 2 × 10^7^ paired reads per sample with read length of 125 × 2 bp. Individual transcripts are depicted as individual squares. Transcripts above the *x* axis are upregulated, while transcripts below the *x* axis are downregulated. Transcripts are color coded by transcript type and transcript types are quantified for relative abundance in top right inset pie chart. **d** DETs are enriched for gene ontology terms related to multiple neuronal compartments demonstrating different ways in which genes can regulate cellular function (Bonferoni *p* < 0.05). Several of the genes implicated in regulating these cellular compartments (*Ankr*, *Map2*, *NrCam*) are implicated in regulating neuronal structural and functional plasticity. **e** Cell type-specific expression of GABAergic neuron and other interneuron subtype transcriptional markers. *Npy* neuropeptide Y, *Nos1* nitrous oxide synthase 1, *Lhx6* Lim-containing homeobox 6, *Cck* cholecystekinin, *Pv* parvalbumin, *Chat* choline acetyltransferase, *Vip* vasoactive intestinal peptide

We next sought to understand the molecular adaptations induced in NAc somatostatin interneurons by use of RNA-sequencing. These cells offer a particular challenge to profile transcriptionally because of their minute numbers. To profile the entire (non poly-A selected) transcriptome of NAc somatostatin interneurons, we generated a transgenic reporter line (SST-TLG498 mice) to label the nuclei of these cells with a modified form of EGFP that is retained in the nuclear membrane (EGFP-F)^22^, enabling their isolation from NAc dissections using FACS (Fig. 2a). We succeeded in FACS-isolating nuclei suitable for RNA-sequencing from individual SST-TLG498 mice (Fig. 2b), based on previously published methods (Supplementary Fig. 5)^23–25^. We extracted RNA from sorted EGFP(+) and EGFP(−) nuclei and performed qPCR for *Sst* transcripts, which were strongly enriched in the EGFP(+) fraction and barely detectable in the EGFP(−) fraction (Student’s *t*-test: EGFP(+) vs. EGFP(−), *n* = 3, *P* < 0.0001).

After validating our sorting method, we performed RNA-sequencing on EGFP(+) nuclei from a separate group of 20 individual SST-TLG498 mice that received 7 daily IP injections of either saline or cocaine (7.5 mg/kg). We observed increased locomotor activity in cocaine-treated SST-TLG498 mice (Supplementary Fig. 6). The resulting RNA-sequencing data were of high quality (Fig. 2c, d). This is evidenced by the wide distribution of transcript types that we identified and the high mapping rates within each sample. We also observed a small percentage of rRNA reads in our samples compared with other non-coding RNA transcript types, indicating that nuclear sorting can be used to sequence total RNA without bias for rRNA. Therefore, our data better represent dynamic transcriptional regulation that is partly lost in whole tissue or cell preparations where nuclear RNA is mixed with cytoplasmic steady-state mRNAs. We used the nuclear RNA-sequencing data to further confirm the cell type-specificity of our sorted cell population (Fig. 2e). *Sst* transcripts, along with transcripts for several other molecules that are known to be expressed by somatostatin interneurons—*Nos1*, neuropeptide Y (*NPY)*, and the canonical MGE-derived post-mitotic GABAergic interneuron marker *Lhx6*^4,26^, were all highly expressed. In contrast, markers of other GABAergic neuron subtypes or of NAc cholinergic interneurons were barely detectable (Fig. 2e).

We proceeded with differential expression analysis of the RNA-sequencing data to identify differentially expressed transcripts (DETs) in NAc somatostatin interneurons in response to repeated cocaine exposure: 778 transcripts were upregulated by cocaine and 322 were downregulated (Fig. 2c). These included many differentially expressed non-coding RNAs of each transcript type, with the frequency of transcript types among DETs being similar to those in the whole transcriptome. We succeeded in identifying a DET signature for cocaine action: all mice in the cocaine arm are differentiated from all mice in the saline arm solely by unsupervised clustering of the expression values from the 75 most robust DETs (logFC, *p* < 0.05) (Supplementary Fig. 7). Thus, we have high confidence that the DETs we identified are genuinely regulated by cocaine, despite individual mouse variability in specific transcript expression levels.

Database for annotation, visualization and integrated discovery (DAVID) was used to identify the functional gene ontology (GO) of the 1100 DETs in NAc somatostatin interneurons. We used this strategy to determine if a specific cellular sub-compartment was the target of cocaine-induced transcriptional alterations in somatostatin interneurons—for example, whether the differentially expressed genes are only enriched for proteins that function within synapses. On the contrary, we found that 8 cellular sub-compartments were significantly enriched in our DET dataset that are specific to neurons (Bonferoni *p* < 0.05) (Fig. 2d). Underlying these GO terms are changes in transcripts encoding proteins that regulate cytoskeletal architecture and cellular adhesion: ankryn (*Ankr*), neuronal cell adhesion molecule (*NrCam*), and microtubule-associated protein 2 (*Map2*), which have well-established roles in structural and functional plasticity^27–29^. Our analysis also identified several additional differentially expressed protein-coding genes, e.g., calcium/calmodulin-dependent protein kinase II inhibitor 1 (*Camk2n1*) and several transcription factors—*JunD*, forkhead box O3 (*FoxO3*), and nuclear factor kappa-light-chain-enhancer of activated B cells (*NF-κB*), all implicated previously in cocaine-induced plasticity in NAc^30–33^. However, it is important to note that these earlier studies relied on methods that manipulated and studied all NAc neurons using whole tissue lysates, which we avoid here by selectively studying somatostatin interneurons. The finding of *JunD* transcriptional regulation in particular drew our interest, as it is an AP-1 family transcription factor that has been implicated as a binding partner for ∆FOSB in principal MSNs of the NAc^30,34^. Our new results therefore raise the important question as to what role JUND may have in somatostatin interneurons, where ∆FOSB is not induced by cocaine, as these cells constitute a miniscule fraction of all cells contained in whole NAc homogenates.

### Cocaine alters JUND in NAc somatostatin interneurons

Our RNA-sequencing data revealed that *JunD* transcription was upregulated in NAc somatostatin interneurons after repeated cocaine exposure (*JunD*: Saline = 1.75 logCPM vs. Cocaine = 4.04 logCPM, logFC = 2.58, *p* < 0.05). We used the ENRICHR platform to determine transcription factors that may be upstream of highly expressed cocaine-altered transcripts, based on known transcription factor protein–protein interactions^34,35^. JUND was also predicted as upstream of the DETs in our dataset (Expression cutoff logCPM > 1; JUND: *p* < 0.05). Both lines of evidence support the hypothesis that JUND may be a critical factor in mediating cocaine-induced alterations in somatostatin interneurons in the NAc.

**Fig. 3:**
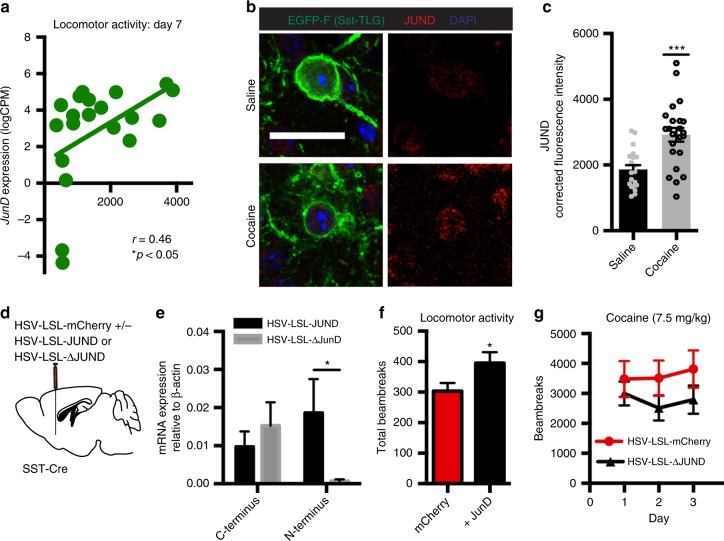
*JunD* expression in NAc somatostatin interneurons regulates locomotor behavior. **a** There is a positive correlation in our RNA-sequencing data between *JunD* expression levels and locomotor activity across saline- and cocaine-treated mice on the last day of testing (Pearson Correlation: *r* = 0.46, *n* = 20, *p* = 0.043). Green circles represent *JunD* expression levels from individual mice. **b** Representative images used for quantification of JUND corrected total cell fluorescence (CTCF) in EGFP(+) cells. Each image is a single plane in a z-stack to ensure true fluorescence signal (not the sum of multiple planes). Scale bar = 25 µm. **c** JUND protein expression is increased in EGFP(+) cells of cocaine-treated mice (1 h after last dose) compared to saline controls (Student's *t*-test with Welch’s Correction: ***p < 0.0001; Saline mean CTCF = 1807 ± 138.9 SEM (*n* = 20 cells/3 mice) vs Cocaine mean CTCF = 2920 ± 207.7 SEM (*n* = 23 cells/3 mice). Welch’s correction: t = 4.54, df = 37.77). **d** Stereotaxic targeting of HSV-LSL-JUND + HSV-LSL-mCHERRY or HSV-LSL-ΔJUND + HSV-LSL-mCHERRY in NAc of *Sst-Cre* mice. **e** qPCR quantification of transgene expression in NAc of *Sst-Cre* mice infected with HSV-LSL-JUND or HSV-LSL-mCHERRY. ∆*JunD* is a truncated form of *JunD* that lacks the N-terminus, making it a dominant-negative protein. (Student’s *t*-test with Welch’s correction: *p < 0.05; HSV-LSL-JUND mean = 0.01859 ± 0.008903 SEM (*n* = 8) vs HSV-LSL-ΔJUND mean = 0.0007039 ± 0.0004589 SEM (*n* = 10) Welch’s Correction: *t* = 2.259, df = 16). **f** *JunD* overexpression in NAc somatostatin interneurons increases locomotor activity in an open field (Students *t*-test: *p = 0.051; mCherry mean = 303.4 ± 26.25 SEM vs + *JunD* mean = 395.5 ± 35.62 SEM (*n* = 11,11)). **g** Dominant-negative*ΔJunD* expression in NAc somatostatin interneurons decreases locomotor responses to cocaine (Repeated Measures Two-way ANOVA: Significant effect of Virus F _(1,20)_ = 4.937. *p* < 0.04; *n* = 10,10). Data are represented as ± SEM

Consistent with this hypothesis, we found that *JunD* expression levels in NAc somatostatin interneurons from individual mice were correlated with locomotor activity on the final day of testing regardless of cocaine or saline exposure (Fig. 3a). To study cocaine regulation of JUND protein expression in NAc somatostatin interneurons, we performed immunohistochemistry for JUND in saline- and cocaine-treated SST-TLG498 mice. We determined first that cocaine did not alter the percentage of EGFP(+) cells that were immunoreactive for JUND. Next, we measured the total corrected cell fluorescence of JUND in individual NAc somatostatin interneurons and found that JUND levels in individual cells were increased in cocaine-treated mice compared to saline controls (Fig. 3b, c). In contrast, JUND levels are not altered by cocaine exposure in non-somatostatin cells—i.e., EGFP(−) cells—in NAc despite being expressed broadly in these cells at baseline (Supplementary Fig. 8). This result confirms prior interpretations that while JUND functions in MSNs as a binding partner for ∆FOSB, it itself is not regulated by cocaine in that cell type^30,34^. Together, these findings support the RNA-sequencing finding that *JunD* expression is induced selectively in NAc somatostatin interneurons in response to repeated cocaine injections.

To test the behavioral relevance of such *JunD* regulation, we developed a conditional herpes simplex virus (HSV) vector to overexpress *JunD*, or its truncated, dominant-negative mutant termed *∆JunD*—previously validated in vitro and in vivo^36^, selectively in NAc somatostatin interneurons. Because *∆JunD* is a truncated form of *JunD*, lacking the N-terminus of the wild-type protein, we were able to use qPCR targeting the C- and N-termini of the *JunD* transcript to validate viral-mediated overexpression of *JunD* versus *ΔJunD* in NAc of *Sst-Cre* mice (Fig. 3d, e). We found that NAc of mice treated with HSV-JUND exhibit an equal proportion of C- and N-termini consistent with full-length *JunD* overexpression, whereas mice treated with HSV-∆JUND exhibit greater expression of C-terminus *JunD* consistent with *ΔJunD* lacking the N-terminus of the wild-type transcript.

Using this HSV system we found that *JunD* overexpression in NAc somatostatin interneurons increased baseline locomotor activity of mice in an open field (Fig. 3f). However, we did not observe an increase in locomotor activity in response to cocaine after *JunD* overexpression or an effect on cocaine CPP (Supplementary Fig. 9). This could be due to high levels of JUND protein expressed by these neurons especially after cocaine exposure or a lack of temporal control over *JunD* transcription that may occur endogenously (see Discussion). Conversely, we found that overexpression of *ΔJunD* in NAc somatostatin interneurons decreased locomotor responses to repeated doses of cocaine, as would be expected from our RNA-sequencing experiment (Fig. 3g).

### Cocaine alters NAc somatostatin interneuron excitability

**Fig. 4:**
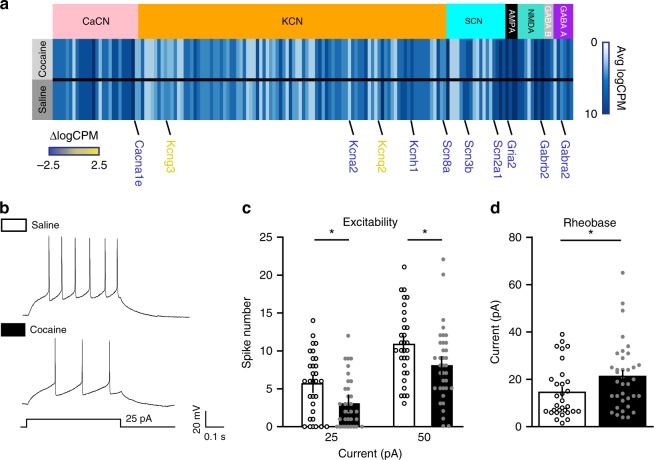
The excitability and rheobase of NAc somatostatin interneurons are altered by repeated cocaine exposure. **a** Expression profiling of neuronal ion channels in NAc somatostatin interneurons. Average expression of each transcript is shown as a vertical line for each treatment group with blue intensity representing level of expression. White colored transcripts are lowly or not expressed whereas dark blue transcripts are more highly expressed. Significant changes in the transcription of specific channels after cocaine are shown under the heat map with the change in expression depicted by the font color of the gene name, such that yellow represents increased expression, while blue indicates decreased expression. **b** Representative traces of spike number obtained by 25 pA current injection in EYFP(+) NAc somatostatin neurons, as a measure of intrinsic neuronal excitability. **c** Whole-cell quantification of current-induced spike numbers in EYFP(+) NAc somatostatin neurons showed that repeated cocaine administration resulted in decreased cellular excitability with 25 and 50 pA current injections as compared to saline-treated controls (Two-way ANOVA, Bonferroni posthoc-test: cocaine treatment F_(1, 65)_ = 7.02, *p* = 0.0101; current injection F_(1, 65)_ = 269.89, *p* < 0.0001; interaction F_(1, 65)_ = 0.10, *p* = 0.7561; *n* = 31–36 cells per group, 5 mice per group). **d** Whole-cell quantification of current injection-induced first spike numbers in EYFP(+) NAc somatostatin neurons showed that increased current was needed to induce the first spike (rheobase) in repeated cocaine-treated mice compared to saline controls (Gaussian fit (Shapiro–Wilk) normality test *p* < 0.05, two-tailed Mann–Whitney test *p* = 0.0415; *n* = 31–36 cells per group, 5 mice per group). Data are represented as ± SEM

Along with changes in transcripts related to transcriptional and structural plasticity, we observed cocaine-induced alterations in the expression levels of many types of ion channels in NAc somatostatin interneurons (Fig. 4a). Changes in the expression of these transcripts contributed to several identified GO terms (Fig. 2d). We therefore investigated whether the cellular excitability of NAc somatostatin neurons was altered by repeated cocaine exposure. First, we injected *Sst-Cre* mice with AAV-DIO-EYFP and performed recordings from EYFP(+) cells in brain slices of the NAc. The cells from which we recorded exhibited similar baseline properties previously seen in striatal somatostatin interneurons^20,37^. We found that cocaine does not alter the resting membrane potential of these cells (Cocaine: −59.19±0.64 mV; Control: −59.94±0.59 mV; Mann–Whitney test, *p* = 0.38; *n* = 31–36 cells and 5 mice per group). However, cocaine decreased the number of evoked action potentials in response to current injections compared to saline treatment (Fig. 4b, c). Consistent with this decreased neuronal excitability, we also observed an increased threshold to induce the first spike (rheobase) in response to current injections in the cocaine-treated group (Fig. 4d). These results show that repeated cocaine exposure decreases the excitability of NAc somatostatin interneurons.

## Discussion

In this study, we used complementary methods to characterize the role played by NAc somatostatin interneurons in molecular and behavioral responses to cocaine. Using RNA-sequencing, we obtained a total transcriptome map of gene expression changes elicited in NAc somatostatin interneurons by repeated cocaine administration, and used these novel cell type-specific data to identify a previously unappreciated role for JUND acting in this cell population in cocaine action. We also found that repeated cocaine reduces the intrinsic excitability of NAc somatostatin interneurons and, using optogenetics, that cocaine-induced locomotor activity and reward are dependent on the activity of these neurons, despite their sparse numbers in NAc.

Our finding that optogenetic activation of NAc somatostatin interneurons promotes cocaine responses raises the question as to which neurotransmitter is responsible, given that somatostatin interneurons synthesize and release GABA, SST, and NPY, among other signaling molecules such as NO^5,38^. Somatostatin interneurons are known to directly influence distal dendrites of principal MSNs via GABAergic signaling^7,20^. Previous studies have shown that infusion of somatostatin peptide alters dopamine release in striatum and that somatostatin infusion alters amphetamine-induced locomotor activity in rodents, suggesting that somatostatin signaling is also involved^10,13^. Nevertheless, it is likely that this population of cells utilizes multiple neurotransmitter systems each of which alters the physiology of NAc microcircuits^3^. Future studies using optogenetics in combination with microfluidic infusion of specific receptor antagonists will eventually be able to systematically address this question.

We used a nuclear sorting method by which cells in frozen tissue are lysed rapidly, enabling the effective isolation of nuclei from other cellular organelles in a manner that avoids the use of enzymes to dissociate the tissue. As transcription of new genes requires ATP, it is unlikely that isolated nuclei possess the energetic requirements to induce transcription during FACS. Importantly, our data showing minimal expression levels of *c-Fos* transcripts in NAc somatostatin interneuron nuclei isolated from saline-treated mice are in line with recent reports showing little or no *c-Fos* activation in sorted nuclei from naive mice in contrast to sorted whole cells which display high levels of *c-Fos*^25^. This is a critical consideration for the field moving forward, as transcriptional changes induced by FACS isolation of live whole cells may confound cell type-specific RNA-sequencing data. Nuclear sequencing thus offers an advantage with the understanding that it captures a distinct population of transcripts.

We identified several differentially expressed protein-coding genes that warrant future study in somatostatin interneurons (Supplementary Data 1). In particular, our sequencing results pointed toward *JunD* expression in NAc somatostatin interneurons as influencing behavioral responses to cocaine. Using immunohistochemistry, we found that cocaine increases JUND expression in somatostatin interneurons, but not in principal neurons, in the NAc. We used viral gene transfer to bidirectionally manipulate *JunD* activity selectively in NAc somatostatin interneurons and found that *JunD* overexpression increased baseline locomotor activity, while the dominant-negative *∆JunD* mutant blunted locomotor responses to cocaine. The relatively high baseline expression of JUND in these neurons, and the further induction of JUND by cocaine, may contribute to the inability to modify behavioral responses to cocaine with *JunD* overexpression. Another possible explanation for the lack of effect of *JunD* overexpression on locomotor or CPP responses to cocaine relates to the temporal features of *JunD* regulation. Our RNA-sequencing and immunohistochemistry data show induction of JUND expression in NAc somatostatin interneurons 1 h after the last dose of cocaine, while our HSV overexpression system induces sustained induction of the protein over several days. It is possible that pulsatile regulation of *JunD* activity would exert more potent behavioral effects. In any event, we show a positive correlation between *JunD* transcription and locomotor activity, which together with our behavioral data highlight *JunD* as a novel cell type-specific target for future study.

Based on the regulation of large numbers of ion channels in NAc somatostatin interneurons following repeated cocaine administration, we studied whether cocaine alters the excitability of these cells and indeed established a cocaine-induced decrease in their intrinsic excitability. Our observation that optogenetic suppression of these cells blunts behavioral responses to cocaine, while activation of the neurons exerts the opposite effect, suggests that cocaine-induced adaptations in NAc somatostatin interneurons are homeostatic, namely, that they have the net effect of dampening cocaine responses. Such tolerance to the behavioral effects of cocaine have been observed both in human addicts and in animal models^39–41^, and several molecular and cellular adaptations to repeated cocaine exposure have been previously shown to mediate such homeostatic functions.^42,43^ The results of this study thereby provide new insight into designing treatments for cocaine addiction that target this small but critical population of NAc interneurons.

## Methods

### Animals and drugs

*Sst-IRES-Cre(*+*/*+*)* mice (*Sst-Cre*; Jackson Labs: *Ssttm2.1(CRE)Zjh/J* Stock –013044) were crossed with *ROSA-TLG498* (TLG498) conditional reporter mice^22^ which express EGFP-F, a hydrophobic variant of GFP that intercalates in the nuclear and other cellular membranes, in *Sst* expressing cells. Male 8–12-week-old *Sst-Cre* mice or *Sst-Cre x TLG498* (SST-TLG498) mice, all on a C57BL/6 J background, were fed ad libitum and housed at 22–25 °C on a 12 h light/dark cycle. All experiments were performed in accordance with guidelines from the Society for Neuroscience and the institutional animal care and use committee at Mount Sinai. All mice were acclimated to vivarium conditions for at least 1 week prior to experimental manipulations. Sample sizes were constrained by the availability of transgenic mice but were ultimately determined based on our prior studies. For all experiments, cocaine-HCl (Sigma) was dissolved in sterile saline and administered via intraperitoneal injection.

### Immunohistochemistry

Immunohistochemical experiments were performed at room temperature in 2 ml microcentrifuge tubes on a rocking plate. All experiments used the same blocking buffer that consists of 0.1% Triton in phosphate-buffered saline and 3% bovine serum albumin (BSA). All sections were mounted using Prolong Gold Antifade (Life Technologies) and kept at 4 °C for short-term storage and −20 °C long-term storage. For SST staining, Rabbit Anti-Somatostatin from Santa Cruz (sc-55565) was used. For JUND staining, Rabbit Anti-JUND from abcam (ab134067) was used. For C-FOS staining, Rabbit Anti-C-FOS from Santa Cruz (sc-52) was used.

### Quantitative polymerase chain reaction (qPCR)

cDNA was generated using iScript cDNA synthesis kit. qPCR was then performed using the 7900HT qPCR system (Thermo Fisher). CT values were normalized to GAPDH, which was not affected by cocaine exposure. Primers used include: *Sst* – FWD: AAGGAAGATGCTGTCCTGCC, REV: TTGGCCAGTTCCTGTTTCCC; *JunD* C-Terminus – FWD: TTCTATGGCGAGGAGGCTCT, REV: GAGCTCCAGGGAAAGCTCTG; *JunD* N-Terminus – FWD: CTGGTGACCACCACACCTAC, REV: CGGCAAATTCCTGCTCTTCG.

### Nuclear FACS isolation from individual SST-TLG498 mice

Nuclei were isolated from SST-TLG498 NAc punches of individual mice and prepared for FACS with modifications to established methods^23,24^. Triton X-100 was omitted from all buffers to maintain EGFP-F in the nuclear membrane. Lysed cellular extracts were spun on a sucrose gradient in an ultracentrifuge at 24.4 K RPM at 4 °C for 1 h with a swing bucket rotor. Nuclei were triturated and vortexed into a suspension and filtered through a 35 µm mesh. Wild-type mice were used to establish gating strategy on a FACS machine before transgenic samples were analyzed. On average, EGFP-F + nuclei accounted for a small but replicable percentage (1–2%) of the nuclei in each set of punches, consistent with previous histological estimates^3^. EGFP-F + nuclei were sorted through 100 µm nozzle at 20 PSI and collected in 750 µl of Trizol LS. Immediately after sorting, collection tubes were vortexed and flash frozen on dry ice. Samples were stored frozen in Trizol LS at −80 °C until RNA extraction and library preparation.

### RNA isolation and library preparation for total RNA-sequencing

RNA was extracted directly from Trizol LS using Directzol RNA-Mini prep (Zymo). Samples were exposed to DNAse for 15 min as per kit protocol. RNA was eluted into 12 µl H2O to ensure viable concentration of RNA for 8 µl library prep input volume limit. Libraries were prepared for paired-end sequencing on HiSeq 2000 using Takara (Formerly Clontech) Smarter Stranded Total RNA-seq Kit (Cat #: 634838) and validated using Agilent Bioanalyzer, Agilent Tapestation, and PCR prior to sequencing.

### Bioinformatics analyses

Raw 100 bp cDNA reads were aligned to the Ensembl GRCm38.75 mouse reference genome via STAR. Raw read count values were deduced from quantitation against the Ensembl GRCm38.75 annotation features using featureCounts from the Subread package^44^. These raw count values were normalized by the weighted trimmed mean of M-values, which scales the samples by the raw library sizes. This normalized gene expression matrix used was used to study differences in gene expression between cocaine- and saline-treated groups. Differential expression was estimated using the Voom method and genes were considered differentially regulated at a nominal *p*-value threshold of 0.05.

### Viral vectors and stereotaxic surgery

AAV-Ef1a-DIO-CHR2(H134R)-EYFP or AAV-EF1A-DIO-EYFP were obtained from the UNC viral vector core facility.

*JunD* and *∆JunD* were mouse codon optimized, de novo synthesized and sequence-validated. They were individually sub-cloned into bi-cistronic HSV vectors that express GFP under the *CMV* promoter. The transgenes were inserted following a stop codon surrounded by loxP sites driven by the IE 4/5 promoter allowing expression of GFP in all transduced cells and expression of our constructs only in cells also expressing *Cre* recombinase (i.e., somatostatin interneurons). Expression of HSV transgenes is maximal by 1–5 days after infusion. qPCR and immunohistochemistry were used to validate targeting, specificity, and expression of the transgenes. qPCR primers were made targeting either the C-Terminus or N-terminus of endogenous *JunD* protein. Since *∆JunD* lacks the N-terminus of endogenous *JunD*, we expect to see more qPCR product with the N-terminus primers in NAc of mice injected with HSV-LSL-JUND compared to HSV-LSL-∆JUND.

Mice were anesthetized with ketamine (100 mg/kg) and xylazine (10 mg/kg) in sterile saline and administered by intraperitoneal injection. Thirty-three gauge needles were used to bilaterally infuse AAV or HSV vectors into NAc (AP = 1.5, ML = ± 1.5, and DV = −4.4; 10° angle). An infusion volume of 0.5 μl was delivered using a 5 μl Hamilton syringe over the course of 5 min (at a rate of 0.1 μl/min). The infusion needle remained in place for at least 5 min after the infusions before removal to prevent backflow of the viruses. For optogenetics experiments, custom-made optical ferrules (Doric Lenses) with fibers 3.9 mm in length from the base of the cannula were implanted up to 3 weeks after viral injection targeting the NAc (AP = 1.5; ML = 1.3; DV = –3.9; 0° angle) and mice were allowed to recover for at least 1 day before behavioral experiments.

### Cocaine-induced locomotor activity in SST-TLG498 mice

A population of 20 individual SST-TLG498 mice were exposed to either daily saline or cocaine injections (7.5 mg/kg) for 7 days and locomotor activity was assessed^21^. Briefly, activity was assessed in rat-sized cages within a locomotor apparatus and mice were evaluated for horizontal ambulation in the *x* and *y* planes by quantifying laser-grid beambreaks with Med Associates software. An hour after the final injection of saline or cocaine, the mice were euthanized by decapitation and individual brains were extracted. Brains were immediately sectioned and a 200 µm thick section containing NAc was obtained. 14 gauge blunted needles were used to collect bilateral tissue punches of NAc from individual mice, which were immediately frozen on dry ice and stored at −80 °C until the day of FACS.

### Cocaine-induced locomotor activity with optogenetic manipulation of NAc somatostatin interneuron activity

Locomotor activity was performed in the following manner using rat-sized cages set up in locomotor apparati such that mice were assessed for movement on an *x–y* plane as measured by laser-grid beambreaks with Med Associates software. On day 1–5, pre-made optical fibers (Doric Lenses) were secured bilaterally to the fiber optic cannula (Doric Lenses) and the mice were allowed to habituate to the test environment for 5 min prior to drug administration and light manipulation. Then, either saline or cocaine (7.5 mg/kg IP) was administered and the light function generator was activated to induce “Stimulation” (473 nm, 20 Hz frequency, 1 burst of 10 pulses, 4 ms pulse width, every 10 s) or “Suppression/Depolarization Block” (473 nm, 20 Hz frequency, 1 burst of 20 pulses, 49 ms pulse width, every sec) in somatostatin interneurons adapted from previous studies^17^. Both stimulation and suppression were validated in brain slices (Supplementary Fig. 2). Control EYFP mice were also exposed to laser manipulations. Locomotor activity was assessed for 30 min on each day while being paired with optogenetic manipulation. Mice were perfused 1 h after the final injection of cocaine on day 7.

### CPP with optogenetic manipulation of NAc somatostatin interneuron

An unbiased CPP paradigm utilizing Med Associates software and 3-chamber CPP boxes. The boxes consist of a small middle chamber, and two larger chambers on eat side. The larger conditioning chambers include different contextual cues (i.e., different spacing on wire grid flooring, different colored striped walls). *Sst-Cre* mice infected with AAV-DIO-EYFP or AAV-DIO-CHR2-EYFP were placed in a three-chamber CPP box for a 20 min “pretest” to ensure that groups had no chamber bias; <10% of all mice show a bias defined as 100 s. Groups were then balanced to adjust for any chamber bias that remained between the groups. In total, a small number of mice with pre-existing bias >600 s were excluded from the studies prior to training. Mice were then trained for 2 days to cocaine (2.5, 5 or 7.5 mg/kg) without or with optogenetic stimulation or suppression or to optogenetic manipulations alone. For optogenetics experiments, optical fibers were not attached during the pretest or test days. During training fiber optic patch cords were secured to the cannulas immediately following injection of either saline or cocaine. In the first training session, mice were injected with saline and confined to one side of the apparatus during which time they were connected to fiber optics but the lasers were not turned on. In the second session, lasers were turned on after cocaine injections and mice were confined to the opposite chamber. Cocaine was only given in the second session to ensure that no cocaine was present in the mice during saline pairing. The experiments were counterbalanced to ensure that cocaine was paired evenly in both sides of the chambers. After two training days, mice were tested and CPP scores determined. CPP scores represent: (time spent in the Cocaine/Optogenetics paired chamber) − (time spent in the unpaired chamber).

### Ex vivo slice validation of optogenetic stimulation protocol in NAc somatostatin interneurons

Three weeks after viral injections, the mice were perfused transcardially with ice-cold oxygenated artificial cerebrospinal fluid (ACSF) which contained 128 mM NaCl, 3 mM KCl, 1.25 mM NaH_2_PO_4_, 10 mM D-glucose, 24 mM NaHCO_3_, 2 mM CaCl_2_ and 2 mM MgCl_2_ (oxygenated with 95% O_2_ and 5% CO_2_, pH 7.35, 295–305 mOsm). Acute brain slices containing the NAc were sectioned at 250 µm in cold sucrose ACSF, which was derived by replacing NaCl with 254 mM sucrose. Slices recovered at 37 °C for 1 h in oxygenated ACSF. Somatostatin cells infected in vivo with the CHR2-expressing AAV were identified via fluorescence visualization. Whole-cell current-clamp recordings were performed at −60 mV, the average resting membrane potential of somatostatin cells in NAc. Glass electrodes (3–5 MΩ) were filled with an internal solution containing 115 mM potassium gluconate, 20 mM KCl, 1.5 mM MgCl_2_, 10 mM phosphocreatine, 10 mM HEPES, 2 mM magnesium ATP, and 0.5 mM GTP (pH 7.2, 285 mOsm). Optic fibers were connected using an FC/PC adapter to a 473-nm blue laser diode (Crystal Laser) and a stimulator (Agilent Technologies, no. 33220A) was used to generate blue light pulses. For ex vivo slice electrophysiological validation of CHR2 activation, we tested a series of burst stimulations to mimic the plateau low threshold spiking patterns observed physiologically in these neurons. Neurons were exposed to 10 spikes (4 ms width) over each 10 s epoch with increasing frequencies.

### Electrophysiological recording of NAc somatostatin interneurons after chronic cocaine

*Sst-Cre* mice were injected with AAV-DIO-EYFP and injected with either saline or cocaine for 7 days in the home cage. Mice were sacrificed 1 h following the final injection of cocaine/saline, as described above. All recordings were carried out blind to the experimental conditions. The anatomical location of the EYFP(+) neurons was validated within the NAc in each recording. Whole-cell recordings were obtained from 250 µm NAc brain slices. Spike number was obtained by 25 pA and 50 pA current injections in 0.5 s increments in current-clamp mode, as a measure of intrinsic neuronal excitability. Whole-cell quantification of current injection-induced first spike numbers was used to determine the rheobase.

### Data availability statement

All of the individual raw RNA-sequencing files are available to the public through the NIH GEO data repository via ascension number: GSE116484

## Electronic supplementary material

Supplementary Information

Description of Additional Supplementary Files

Supplementary Data 1

## References

[1] Lin LC, Sibille E (2013). Reduced brain somatostatin in mood disorders: a common pathophysiological substrate and drug target?. Front. Pharmacol..

[2] Lin LC, Sibille E (2015). Somatostatin, neuronal vulnerability and behavioral emotionality. Mol. Psychiatry.

[3] Tepper JM, Tecuapetla F, Koos T, Ibanez-Sandoval O (2010). Heterogeneity and diversity of striatal GABAergic interneurons. Front. Neuroanat..

[4] Marin O, Anderson SA, Rubenstein JL (2000). Origin and molecular specification of striatal interneurons. J. Neurosci..

[5] Kepecs A, Fishell G (2014). Interneuron cell types are fit to function. Nature.

[6] Stefanelli T, Bertollini C, Lüscher C, Muller D, Mendez P (2016). Hippocampal somatostatin interneurons control the size of neuronal memory ensembles. Neuron.

[7] Chiu CQ (2013). Compartmentalization of GABAergic inhibition by dendritic spines. Science.

[8] Hathway GJ, Humphrey PPA, Kendrick KM (2004). Somatostatin induces striatal dopamine release and contralateral turning behaviour in the mouse. Neurosci. Lett..

[9] Kouvidi E, Papadopoulou-Daifoti Z, Thermos K (2006). Somatostatin modulates dopamine release in rat retina. Neurosci. Lett..

[10] Ikeda H, Kotani A, Koshikawa N, Cools AR (2009). Somatostatin receptors in the nucleus accumbens modulate dopamine-dependent but not acetylcholine-dependent turning behaviour of rats. Neuroscience.

[11] Lopez-Huerta VG, Tecuapetla F, Guzman JN, Bargas J, Galarraga E (2008). Presynaptic modulation by somatostatin in the neostriatum. Neurochem. Res..

[12] Ikeda H, Kamei J, Koshikawa N, Cools AR (2012). Nucleus accumbens and dopamine-mediated turning behavior of the rat: role of accumbal non-dopaminergic receptors. J. Pharmacol. Sci..

[13] Raynor K, Lucki I, Reisine T (1993). Somatostatin receptors in the nucleus accumbens selectively mediate the stimulatory effect of somatostatin on locomotor activity in rats. J. Pharmacol. Exp. Ther..

[14] Santis S (2009). Somatostatin increases rat locomotor activity by activating sst(2) and sst (4) receptors in the striatum and via glutamatergic involvement. N. S. Arch. Pharmacol..

[15] Heiman M (2008). A translational profiling approach for the molecular characterization of CNS cell types. Cell.

[16] Zhang F (2010). Optogenetic interrogation of neural circuits: technology for probing mammalian brain structures. Nat. Protoc..

[17] Herman AM, Huang L, Murphey DK, Garcia I, Arenkiel BR (2014). Cell type-specific and time-dependent light exposure contribute to silencing in neurons expressing Channelrhodopsin-2. eLife.

[18] Veit J, Hakim R, Jadi MP, Sejnowski TJ, Adesnik H (2017). Cortical gamma band synchronization through somatostatin interneurons. Nat. Neurosci..

[19] Hamm JP, Yuste R (2016). Somatostatin interneurons control a key component of mismatch negativity in mouse visual cortex. Cell Rep..

[20] Straub C (2016). Principles of synaptic organization of GABAergic interneurons in the striatum. Neuron.

[21] Lobo MK (2010). Cell type-specific loss of BDNF signaling mimics optogenetic control of cocaine reward. Science.

[22] Chakravarthy S (2008). Cre-dependent expression of multiple transgenes in isolated neurons of the adult forebrain. PLoS ONE.

[23] Matevossian A, Akbarian S (2008). Neuronal nuclei isolation from human postmortem brain tissue. J Vis Exp.

[24] Jiang Y, Matevossian A, Huang HS, Straubhaar J, Akbarian S (2008). Isolation of neuronal chromatin from brain tissue. BMC Neurosci..

[25] Lacar B (2016). Nuclear RNA-seq of single neurons reveals molecular signatures of activation. Nat. Commun..

[26] Petilla Interneuron Nomenclature Group. (2008). Petilla terminology: nomenclature of features of GABAergic interneurons of the cerebral cortex. Nat. Neurosci..

[27] Fanara P (2010). Changes in microtubule turnover accompany synaptic plasticity and memory formation in response to contextual fear conditioning in mice. Neuroscience.

[28] Maas C (2009). Synaptic activation modifies microtubules underlying transport of postsynaptic cargo. PNAS.

[29] De Repentigny Y, Deschênes-Furry J, Jasmin BJ, Kothary R (2003). Impaired fast axonal transport in neurons of the sciatic nerves from dystonia musculorum mice. J. Neurochem..

[30] Chen J (1995). Regulation of delta FosB and FosB-like proteins by electroconvulsive seizure and cocaine treatments. Mol. Pharmacol..

[31] Russo SJ (2009). Nuclear factor B signaling regulates neuronal morphology and cocaine reward. J. Neurosci..

[32] Robison AJ (2013). Behavioral and structural responses to chronic cocaine require a feedforward loop involving ΔFosB and calcium/calmodulin-dependent protein kinase II in the nucleus accumbens shell. J. Neurosci..

[33] Ferguson D (2015). SIRT1-FOXO3a regulate cocaine actions in the nucleus accumbens. J. Neurosci..

[34] Chen EY (2013). Enrichr: interactive and collaborative HTML5 gene list enrichment analysis tool. BMC Bioinforma..

[35] Kuleshov MV (2016). Enrichr: a comprehensive gene set enrichment analysis web server 2016 update. Nucleic Acids Res..

[36] Winstanley CA (2007). DeltaFosB induction in orbitofrontal cortex mediates tolerance to cocaine-induced cognitive dysfunction. J. Neurosci..

[37] Gittis AH, Nelson AB, Thwin MT, Palop JJ, Kreitzer AC (2010). Distinct roles of GABAergic interneurons in the regulation of striatal output pathways. J. Neurosci..

[38] Smith ACW (2017). Accumbens nNOS interneurons regulate cocaine relapse. J. Neurosci..

[39] Koob GF (2009). Neurobiological substrates for the dark side of compulsivity in addiction. Neuropharmacology.

[40] Hammer RP, Egilmez Y, Emmett-Oglesby MW (1997). Neural mechanisms of tolerance to the effects of cocaine. Behav. Brain. Res..

[41] Schenk S, Partridge B (1997). Sensitization and tolerance in psychostimulant self-administration. Pharmacol. Biochem. Behav..

[42] Carlezon WA, Duman RS, Nestler EJ (2005). The many faces of CREB. Trends Neurosci..

[43] Dong Y (2006). CREB modulates excitability of nucleus accumbens neurons. Nat. Neurosci..

[44] Liao Y, Smyth GK, Shi W (2014). featureCounts: an efficient general purpose program for assigning sequence reads to genomic features. Bioinformatics.

