# Supplementary material for: Transcriptional and physiological adaptations in nucleus accumbens somatostatin interneurons that regulate behavioral responses to cocaine
Source: Nat Commun. 2018 Aug 8;9:3149. doi: 10.1038/s41467-018-05657-9 (PMC6082848; doi:10.1038/s41467-018-05657-9)
Supplement: Supplementary file 1 — Supplementary Information [file 41467_2018_5657_MOESM1_ESM.pdf]

Transcriptional and Physiological Adaptations in Nucleus Accumbens Somatostatin Interneurons That Regulate Behavioral Responses to Cocaine

Ribeiro et al.

Supplementary Information

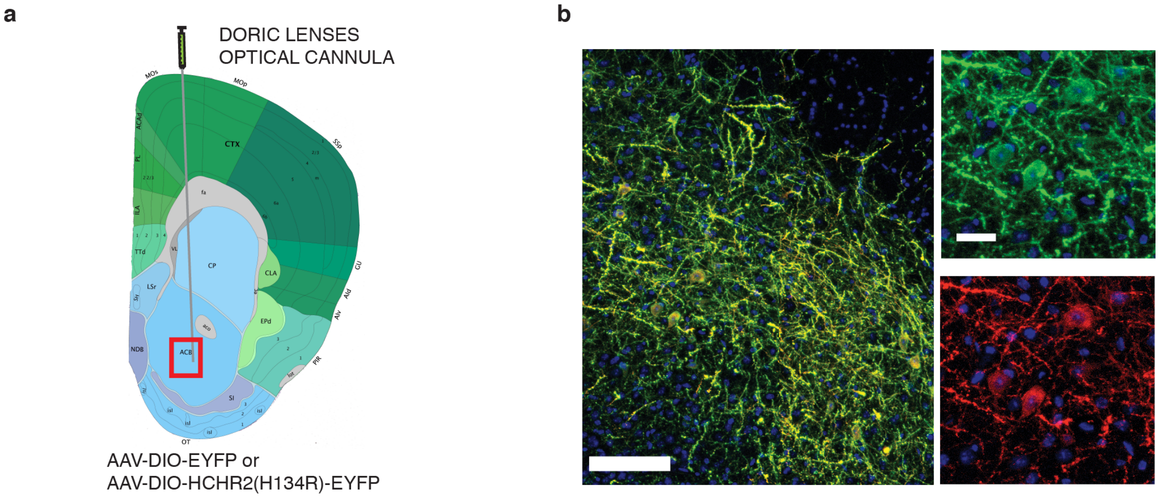

Supplementary Figure 1: Cell type-specific expression of CHR2 in NAc somatostatin interneurons using *SST-Cre* mice. **a**) Anatomical localization of viral injection in NAc shown in B with Red box adapted from Allen Mouse Brain Coronal Atlas image 41/132 (atlas.brain-map.org). ACB – Nucleus Accumbens. **b**) Representative coronal section showing colabelling of SST with EYFP(+) cells infected with AAV-DIO-CHR2-EYFP. SST staining is shown in red. Scale bar = 100μm, inset = 10 μm.

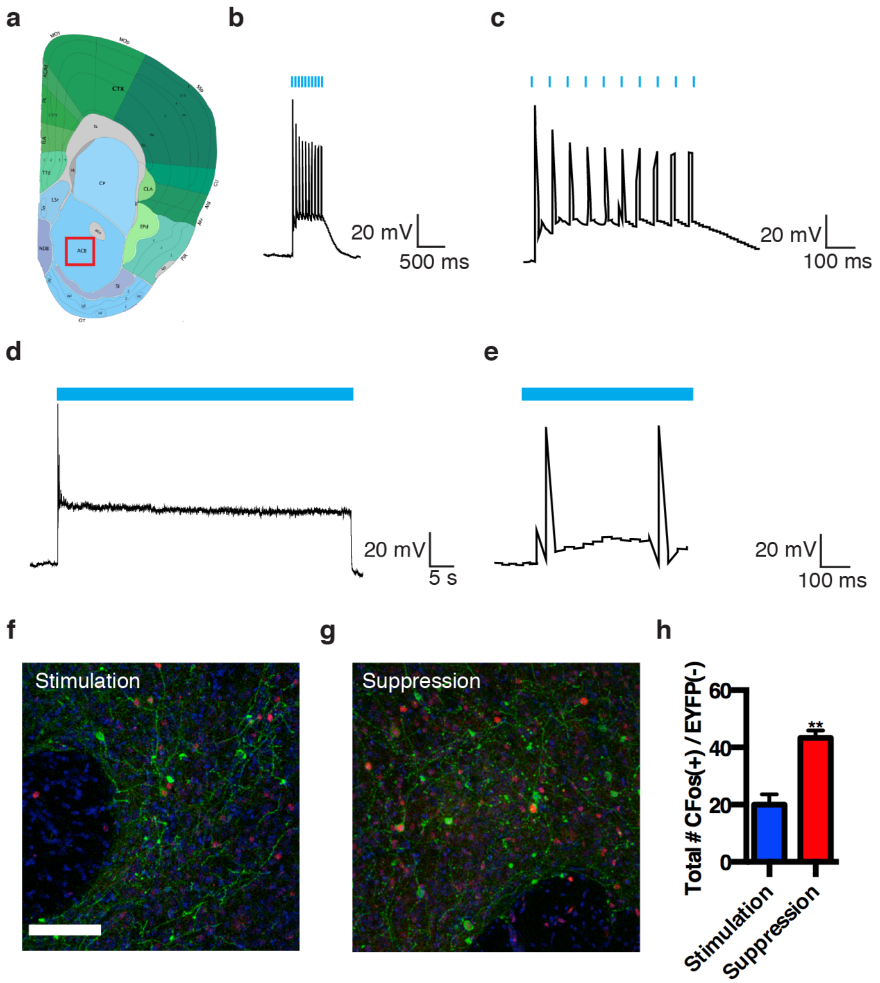

Supplementary Figure 2: NAc somatostatin interneurons can be stimulated or suppressed using CHR2. **a)** Diagram of anatomical location used for ex vivo validation of optogenetic parameters used for somatostatin interneuron stimulation or suppression adapted from Allen Mouse Brain Coronal Atlas image 41/132 (atlas.brain-map.org). ACB – Nucleus Accumbens. Whole cell current clamp recordings were performed on EYFP(+) cells while laser was used to activate CHR2 expressed by somatostatin interneurons within the region defined by red box. **b,c)** Validation of optogenetic parameters used for somatostatin interneuron stimulation using 20 Hz frequency with 4 ms pulsewidth to generate 1 burst of 10 action potentials every 10 seconds based on prior studies[1]. Blue ticks represent 4 ms pulses of blue laser light. **d,e)** Validation of optogenetic parameters used for somatostatin interneuron suppression using 20 Hz frequency but wider pulsewidth (49 ms). This protocol either completely silences the neurons (**d**, n=3 cells/2 mice) or reduces their firing activity to an average of 3.2 Hz (**e**, n=3 cells/2 mice). **d** shows a one minute example of the complete silencing, while **e** shows a 500 ms trace of the reduced firing activity; this time period is equivalent to the 500 ms trace shown for the stimulation protocol in **c**. **f-h)** *C-FOS* (Red) immunohistochemistry following optogenetic modulation of NAc Sst interneuron (EYFP) activity in NAc using Cre-dependent AAV-hChR2. Scale = 100 µm. EYFP staining is specific for somatostatin expressing neurons, thus non-EYFP cells were used to quantify activity of principal neurons following cocaine exposure with optogenetic modulation.

We found that *C-FOS* expression in EYFP(-) principal neurons was increased in mice that received the suppression protocol compared to those that received stimulation (Students t-test: Stimulation vs Suppression, $^{**}p<0.01$; Stimulation Mean=20 ± 3.6 S.E.M. (n=3); Suppression Mean=43.33 ± 2.603 S.E.M. (n=3)). Data are represented as ± S.E.M.

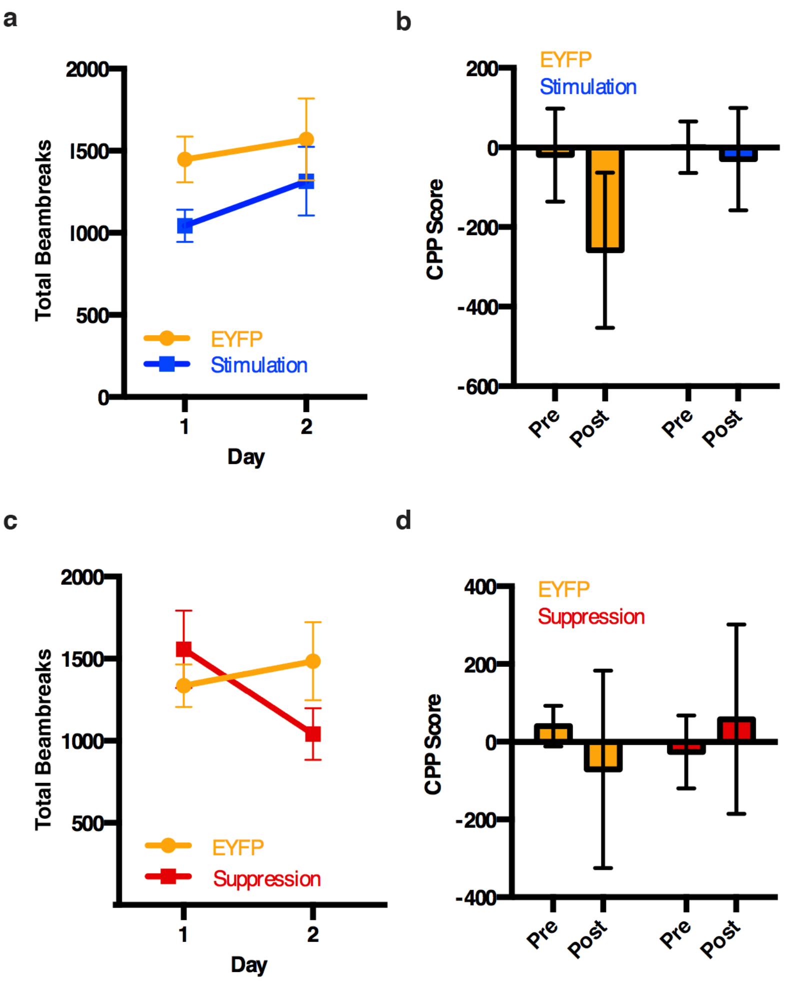

Supplementary Figure 3: Optogenetic manipulation of NAc somatostatin interneuron activity does not alter baseline locomotor activity or conditioned place preference. **a)** Optogenetic stimulation of NAc somatostatin interneurons does not alter baseline locomotor activity. Total beambreaks were quantified over 30 minute sessions. (Students t-test: EYFP vs Stimulation, $p>0.05$; EYFP Mean=524.1 ± 83.13 S.E.M. (n=9); Stimulation Mean=438.2 ± 69.56 S.E.M. (n=9)). **b)** Optogenetic stimulation of NAc somatostatin interneurons alone is neither rewarding nor aversive. The fiberoptic cables were connected during training in both chambers, but lasers were only turned on in one 'paired' chamber. **(**Students t-test: Stimulation Pre-test vs Stimulation post-test, $p>0.05$; Pre-test Mean=0.6364 ± 64.58 S.E.M. (n=11); Post-test Mean=-29.55 ± 128.6 S.E.M. (n=11)**). c)** Optogenetic suppression of NAc somatostatin interneurons does not alter baseline locomotor activity. Total beambreaks were quantified over 30 minute sessions. (Students t-test: EYFP vs Stimulation, $p>0.05$; EYFP Mean=524.1 ± 83.13 S.E.M. (n=9); Stimulation Mean=438.2 ± 69.56 S.E.M. (n=12)). **d)** Optogenetic suppression of NAc somatostatin interneurons alone is neither rewarding nor aversive. Lasers were only turned on in one 'paired' chamber. **(**Students t-test: Stimulation Pre-test vs Stimulation post-test, $p>0.05$; Pre-test Mean=0.6364 ± 64.58 S.E.M. (n=11); Post-test Mean=-29.55 ± 128.6 S.E.M. (n=11)**).** Data are represented as ± S.E.M.

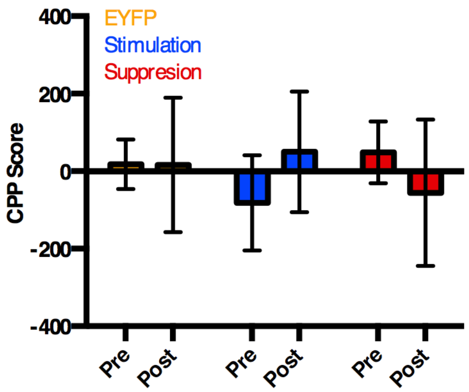

Supplementary Figure 4. Optogenetic stimulation and suppression of somatostatin interneurons does not affect CPP with a sub-threshold dose of cocaine. Training with 2.5 mg/kg cocaine does not induce CPP in control EYFP mice. (Students t-test with Welch's correction: EYFP Pre-test vs Stimulation post-test, $p>0.05$; Pre-test Mean=17.83 ± 63.56 S.E.M. (n=8); EYFP Post-test Mean=16.41 ± 173.5 S.E.M. (n=8))**.** Neither stimulation (Students t-test: ChR2 Stimulation Pre-test vs ChR2 Stimulation post-test, $p>0.05$; ChR2 Stimulation Pre-test Mean=-81.54 ± 122.7 S.E.M. (n=8); ChR2 Stimulation Post-test Mean=-49.85 ± 155.9 S.E.M. (n=8)) nor suppression induces CPP with 2.5 mg/kg cocaine (Students t-test:ChR2 Suppression Pre-test vs ChR2 Suppression post-test, $p>0.05$; ChR2 Suppression Pre-test Mean=48.34 ± 79.62 S.E.M. (n=8); ChR2 Suppression Post-test Mean=-55.68 ± 189.0 S.E.M. (n=8))**.** Data are represented as ± S.E.M.

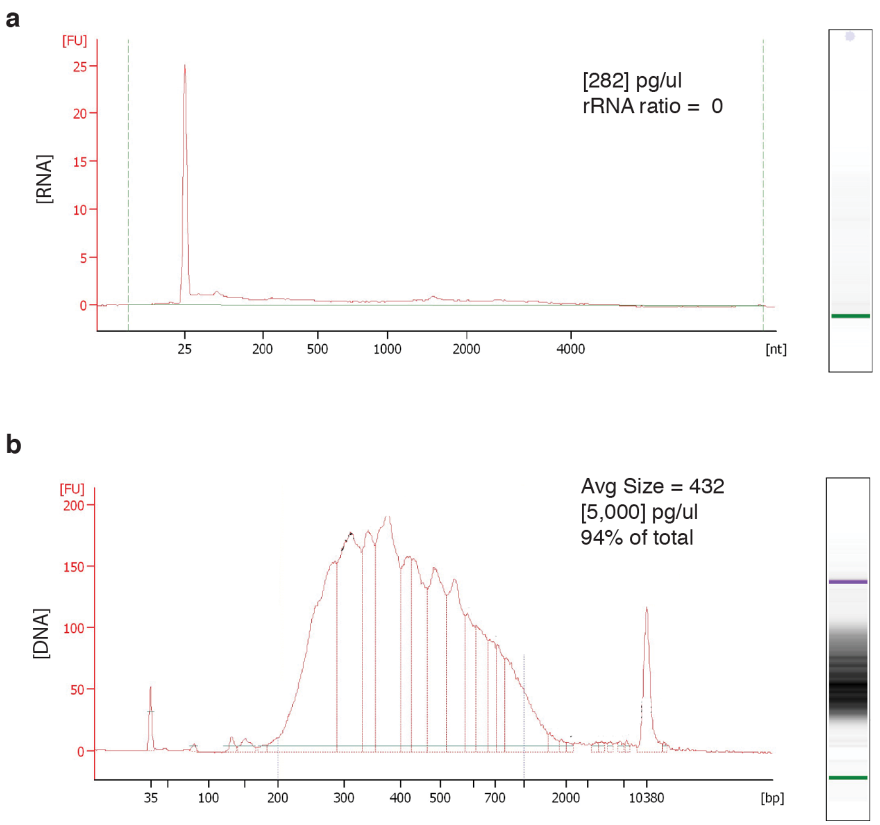

Supplementary Figure 5: Total RNA and resulting library used for RNA-sequencing from individual mice. **a-b**) Representative bioanalyzer traces of 1.5 ng total RNA isolated from nuclei of NAc somatostatin interneurons after FACS and library resulting from Clontech SMARTER Total RNA-Sequencing kit. Note the lack of rRNA in each sample and normal distribution of resulting library from total RNA. Neither ribosomal depletion nor poly-A selection was performed.

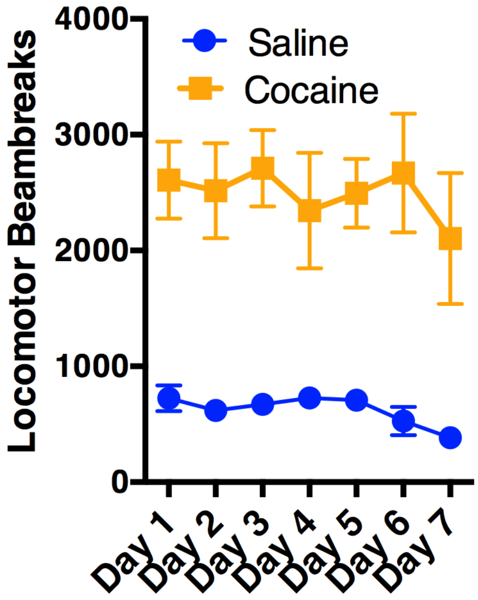

Supplementary Figure 6: Cocaine induced locomotor activity in SST-TLG498 mice used for total RNA sequencing. Cocaine treated mice displayed greater locomotor activity than saline treated mice. (Repeated Measures Two-way ANOVA: Significant effect of Drug $F(1,18) = 64.3$. $p<0.001$, n=10,10).

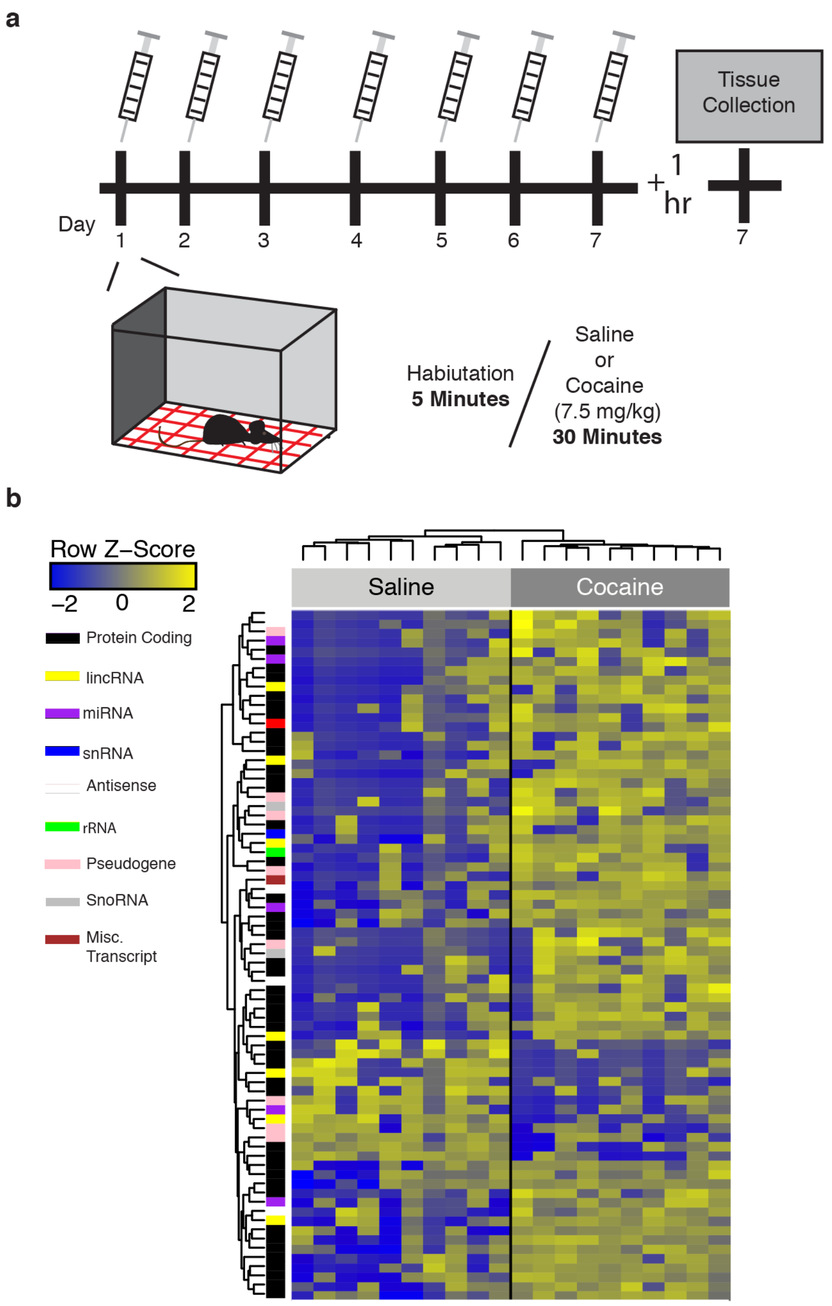

Supplementary Figure 7: Experimental design for identification of cocaine-induced transcriptional changes in NAc somatostatin interneurons. **a**) Timeline of experiment and schematic of locomotor testing chambers used to quantify activity. 10 mice were injected with saline and 10 with cocaine (7.5 mg/kg) every day for 7 days. Mice were used 1 hour after the final injection of cocaine and tissue was flash frozen and stored before RNA extraction. **b**) Cocaine increases locomotor activity in SST-TLG498 mice after repeated cocaine exposure (n=10,10 8 week old males across 5 litters; Repeated Measures Two-way ANOVA: Significant effect of Drug F (1,18) = 64.3. p<0.001). **C)** Unsupervised clustering of samples based on differential expression signature alone. Unsupervised

clustering of DET signature (top 75 logFC difference and $P<0.05$) unambiguously identifies cocaine versus saline treatment. Column Z-scores are represented from blue (negative score/decreased expression) to yellow (positive Z-score/increased expression). Transcript types are shown to indicate diversity of non-coding RNAs captured in signature.

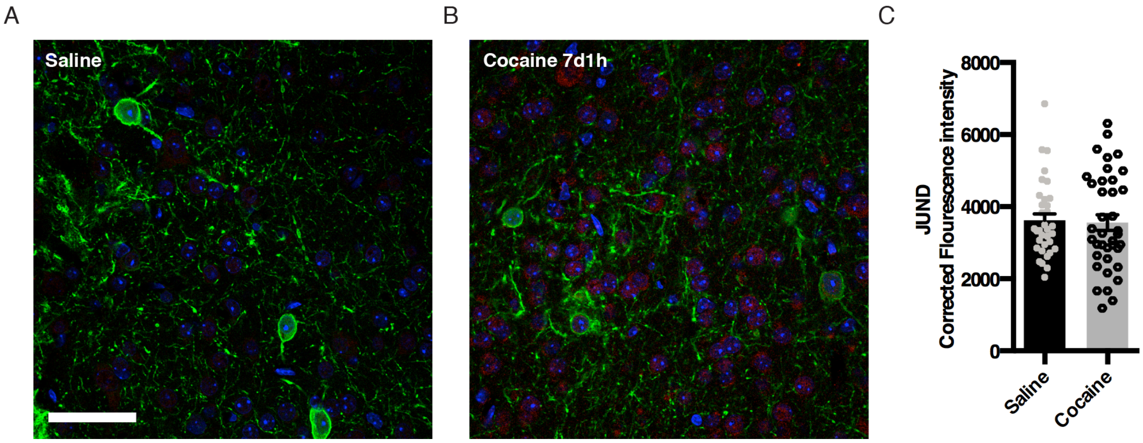

Supplementary Figure 8: JUND expression is not altered by cocaine in non-EYFP principal neurons in the NAc. **a-b)** Immunohistochemical staining for JUND in SST-TLG498 mice used for quantifying JUND intensity in EGFP(-) neurons. Saline and cocaine treatments are shown. Scale bar = 50 μm. **c)** Quantification of JUND corrected fluorescence intensity in EGFP(-) neurons. There is no difference in JUND fluorescence intensity following cocaine administration in these cells. (Students t-test: Saline Mean CTCF=3263 ± 66.65 S.E.M (n= 35 cells/3 mice) vs Cocaine Mean CTCF=3608 ± 101.2 S.E.M. (n=38 cells/3 mice).

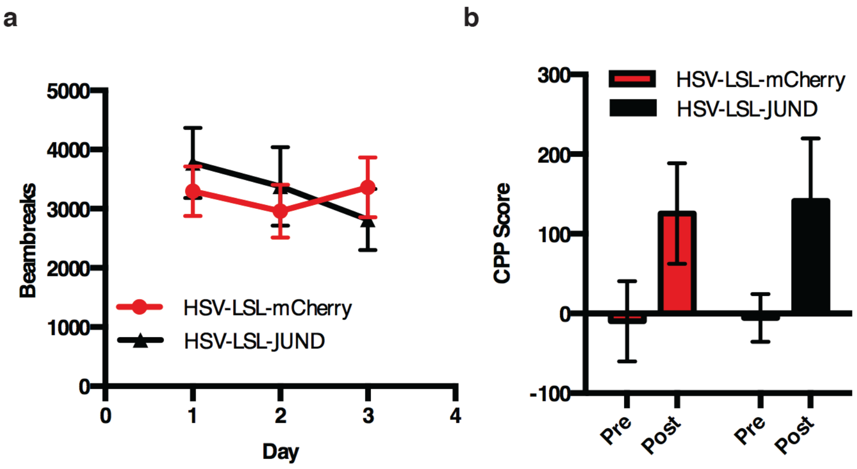

Supplementary Figure 9: Overexpression of *JunD* in NAc somatostatin interneurons does not alter cocaine-induced locomotor activity or CPP. **a**) Overexpression of *JunD* in NAc somatostatin interneurons does not alter cocaine-induced locomotor activity. (Two-way ANOVA: Effect of *JunD* overexpression $F(1,61) = 0.07274$. $p>0.05$, n=11,13). **b**) Overexpression of *JunD* in NAc somatostatin interneurons does not alter CPP to 3.5mg/kg of cocaine. (Students t-test with Welch's correction: HSV-LSL-JUND Pre-test vs post-test, $p>0.05$; Pre-test Mean=-5.610 ± 29.80 S.E.M. (n=11); Post-test Mean=141.4 ± 78.41 S.E.M. (n=11)). Data are represented as ± S.E.M.

Supplementary References

1. Herman, A. M., Huang, L., Murphey, D. K., Garcia, I. & Arenkiel, B. R. Cell type-specific and time-dependent light exposure contribute to silencing in neurons expressing Channelrhodopsin-2. *eLife* doi:10.7554/eLife.01481 (2014).
