# Supplementary material for: Transcriptional and physiological adaptations in nucleus accumbens somatostatin interneurons that regulate behavioral responses to cocaine
Source: Nat Commun. 2018 Aug 8;9:3149. doi: 10.1038/s41467-018-05657-9 (PMC6082848; doi:10.1038/s41467-018-05657-9)
Supplement: Supplementary file 2 — Description of Additional Supplementary Files [file 41467_2018_5657_MOESM2_ESM.pdf]

Description of Additional Supplementary Files

File Name: Supplementary Data 1
Description: This file contains the expression matrix for all 20 individual mice used in our RNA-sequencing experiment. Expression data are expressed as logCPM (Counts Per Million). Transcripts are identified by ensemble mouse genome ID’s (ENSMUSG), gene name and common abbreviation. Transcript type is shown for each transcript. Average expression values are shown for each transcript under both saline and cocaine conditions. deltaCPM represents the change logCPM between saline and cocaine group averages. The log fold-change is shown for each value along with an associated p-value used to determine differential expression. The total transcriptomes from each individual mouse are shown in subsequent columns with expression values in logCPM.
